# Exercise-Induced Oxidative Stress Responses in the Pediatric Population

**DOI:** 10.3390/antiox6010006

**Published:** 2017-01-17

**Authors:** Alexandra Avloniti, Athanasios Chatzinikolaou, Chariklia K. Deli, Dimitris Vlachopoulos, Luis Gracia-Marco, Diamanda Leontsini, Dimitrios Draganidis, Athanasios Z. Jamurtas, George Mastorakos, Ioannis G. Fatouros

**Affiliations:** 1 School of Physical Education and Sport Sciences, Democritus University of Thrace, Komotini 69100, Greece; alavloni@phyed.duth.gr (A.A.); achatzin@phyed.duth.gr (A.C.); dleontsi@gmail.com (D.L.); 2 School of Physical Education and Sport Sciences, University of Thessaly, Karies, Trikala 42100, Greece; delixar@pe.uth.gr (C.K.D.); ddraganidis@pe.uth.gr (D.D.); ajamurt@pe.uth.gr (A.Z.J.); 3 Children’s Health and Exercise Research Centre, Sport and Health Sciences, University of Exeter, Exeter EX1 2LU, UK; dv231@exeter.ac.uk (D.V.); L.A.Gracia-Marco@exeter.ac.uk (L.G.-M.); 4 Growth, Exercise, Nutrition and Development Research Group, University of Zaragoza, Zaragoza 50009, Spain; 5 Faculty of Medicine, Endocrine Unit, “Aretaieion” Hospital, National and Kapodistrian University of Athens, Athens 11528, Greece; mastorakg@ath.forthnet.gr

**Keywords:** exercise, redox regulation, inflammation, childhood, adolescence

## Abstract

Adults demonstrate an upregulation of their pro- and anti-oxidant mechanisms in response to acute exercise while systematic exercise training enhances their antioxidant capacity, thereby leading to a reduced generation of free radicals both at rest and in response to exercise stress. However, less information exists regarding oxidative stress responses and the underlying mechanisms in the pediatric population. Evidence suggests that exercise-induced redox perturbations may be valuable in order to monitor exercise-induced inflammatory responses and as such training overload in children and adolescents as well as monitor optimal growth and development. The purpose of this review was to provide an update on oxidative stress responses to acute and chronic exercise in youth. It has been documented that acute exercise induces age-specific transient alterations in both oxidant and antioxidant markers in children and adolescents. However, these responses seem to be affected by factors such as training phase, training load, fitness level, mode of exercise etc. In relation to chronic adaptation, the role of training on oxidative stress adaptation has not been adequately investigated. The two studies performed so far indicate that children and adolescents exhibit positive adaptations of their antioxidant system, as adults do. More studies are needed in order to shed light on oxidative stress and antioxidant responses, following acute exercise and training adaptations in youth. Available evidence suggests that small amounts of oxidative stress may be necessary for growth whereas the transition to adolescence from childhood may promote maturation of pro- and anti-oxidant mechanisms. Available evidence also suggests that obesity may negatively affect basal and exercise-related antioxidant responses in the peripubertal period during pre- and early-puberty.

## 1. Introduction

Aging, exercise, diseases and other clinical conditions are characterized by a pronounced generation of reactive oxygen and nitrogen species (RONS) which readily oxidize various macromolecules such as lipids, proteins, carbohydrates and nucleic acids [1]. In humans, RONS are neutralized by the antioxidant system which consists of enzymatic (e.g., catalase, glutathione peroxidase (GPX), superoxide dismutase (SOD)) and non-enzymatic molecules (e.g., vitamin E, vitamin C, vitamin A, glutathione, uric acid etc.) [1]. If RONS produced are more than those which the antioxidant system could eliminate, the development of oxidative stress may occur, damaging healthy tissues and cells [2]. Previous research revealed that, in adults, acute exposure to exercise results in marked elevations of oxidative stress and antioxidant status biomarkers with hours to days at rates of various magnitudes depending on intensity, duration, frequency and the degree of eccentric component of the exercise stimulus [2,3,4,5,6,7,8,9,10,11,12,13,14,15,16,17,18,19]. Free radical generation occurs during exercise and post-exercise mainly due to increased activity of xanthine oxidase and nicotine adenine disphosphonucleotide reduced (NADPH) oxidase as well as electron leakage from the electron transport chain in the mitochondria [1,3]. During recovery after exercise, free radicals are produced by neutrophils that infiltrate into the injured muscle fibers as a part of the overall inflammatory response within 2 to 72 h post-exercise [20,21].

Chronic exposure to repetitive generation of RONS in response to systematic exercise (i.e., exercise training) may upregulate antioxidant reserves and reduce acute manifestations of exercise-induced oxidative stress [5,22,23,24,25]. However, in cases of excessive exercise training and inadequate recovery (i.e., overtraining) a disproportionate rise of oxidative stress biomarkers is seen due to the overload of antioxidant system which may be compromised [26,27,28]. It appears that under conditions of elevated oxidant load, ingestion of antioxidant compounds (e.g., thiol-based antioxidants, lipoic acid) [29,30,31,32] and other nutritional supplements [13] may boost antioxidant reserves, help to keep oxidative stress responses under control and lower the inflammatory response. However, it has been suggested that antioxidant reserves, such as reduced glutathione (GSH), may be lower in children, especially those engaged in chronic intensive training programs suggesting that children, and probably adolescents, may be susceptible to exercise-induced oxidative damage [33,34,35]. The higher exercise-induced oxidative stress in children has been linked to the higher oxygen cost of exercise and as such the higher oxygen consumption by skeletal muscles during exercise as well as to age-specific differences of oxidative metabolism [36,37]. Knowledge of exercise-induced oxidative stress responses would be valuable for children and adolescents engaged in systematic strenuous exercise. Moreover, limited information exists about the effects of chronic exercise training on the antioxidant reserves in children and adolescents. Although some evidence suggests that adolescents may benefit from chronic exercise training, data on children’s responses to training is scarce [38]. Furthermore, there is evidence that oxidative stress may be part of the overall growth and development of children, especially during pubertal transition, and exercise training may promote positive adaptations to the anti-oxidant defense system during this transitional period while obesity may negatively impact these adaptations [37,39].

Therefore, the objective of the present review was to present the available data on the unique profile of oxidative stress and antioxidant status responses of children and adolescents. For this review, children are defined as those aged <12 years for studies not reporting stages of sexual maturation or those at stage 1 of the Tanner scale of sexual maturation whereas adolescents are defined as those aged >12 years for studies not reporting stages of sexual maturation or those at stages ≥2 of the Tanner scale of sexual maturation [40]. According to Tanner stages of sexual maturity, a scale that classifies children and adolescents in five stages according to their external primary and secondary sex characteristics, e.g., breast size, genitals, testicular volume and pubic hair [40]. Furthermore, the terms “pediatric population” and/or “youth” refer to both children and adolescents collectively.

## 2. Children Are Not Little Adults

Although, millions of children and adolescents participate in systematic intense exercise training and/or mild physical activities little is known about the acute and chronic effect of different modes of exercise on oxidative and antioxidant responses of this population. Furthermore, it is not correct to use knowledge derived from adult studies in sport applications for children and adolescents.

During developmental stages, children demonstrate changes of smaller magnitude in both intramuscular pH and the ratio of inorganic phosphate to phosphocreatine during exercise, suggesting that they are more dependent on mitochondrial metabolism than adults [33,37]. This adaptation is mainly attributed to children’s lower levels of phosphofructokinase activity (i.e., key-enzyme of the glycolytic pathway) and overall limited glycolytic capacity [41], smaller glycogen stores [27], lower sympatho-adrenal activity [42], different profile of muscle fiber types [43], muscle damage responses of lower magnitude [22] and immature hormonal system [43]. A higher dependence on mitochondrial metabolism to produce energy during exercise in combination with the higher energy cost of exercise in children compared to adults may increase the possibility to generate more RONS during exercise than adults do. Furthermore, a lower production of growth hormone (GH) and testosterone during preadolescence and childhood may predispose children to reduced antioxidant capacity and as such less resistant to free radical generation in response to exercise [39]. However, on the other hand, the fact that children may be less susceptible to skeletal muscle damage in response to exercise than adults may render them more resistant to free radical generation. Recent work has shown that although in children IL-6 and absolute neutrophil count may not rise in response to intense exercise to the degree usually seen in adults, they do exhibit a far greater intracellular neutrophil reactive oxygen species (ROS) generation compared to men, suggesting an age-specific immune response to acute exercise [38]. Thus, children should be considered as unique athletes in terms of exercise-induced responses and not as little adults.

## 3. Responses to Acute Exercise

Although acute exercise effects have been well studied in adults’ population, similar information for the young population is limited. In the field of oxidative stress responses in developmental stages, studies have used various exercise modes, i.e., swimming, cycling, running (steady state and incremental exercise), soccer and basketball.

The Tanner scale (also known as the Tanner stages) is a scale of physical development in children, adolescents and adults. When 70 healthy untrained pre-adolescent (Tanner stage 1, 10–11 years) and adolescent (Tanner stage 2–3, 14 years) boys volunteered to participate in an incremental exercise test of 20-m shuttle run (started at 8 km/h with an increase of 0.5 km/h in every subsequent minute), GSH in saliva demonstrated a marked decline post-exercise compared to its baseline values suggesting that acute exercise in children consumes GSH similar to adults [44]. A more recent study compared the acute responses to intense incremental running of adolescent (Tanner stages 2–3, 14–15 years) and adult track and field athletes [22]. Their results showed that exercise increased the total antioxidant activity (TAC), catalase activity, thiobarbituric acid-reactive substances (TBARS, served as lipid peroxidation marker), protein carbonyls (PC), uric acid, and bilirubin and reduced GSH similarly in adolescent and adult athletes. In this study [22], GSH exhibited similar baseline values among age groups indicating that maturation does not affect its concentration during adolescence. In contrast, an age-dependent post-exercise decline was observed suggesting that maturation affects GSH utilization rates in response to exercise [44]. Therefore, this study provided evidence that running of progressive intensity stimulates the generation of RONS and upregulates antioxidant reserves in an age-dependent manner. Cycling (two sets of 30 min/set with a 6-min rest period between sets) failed to increase protein carbonyls (PC) and malondialdehyde (MDA) in both children (8–10 years) and men [45]. However, following exercise, children demonstrated a greater response in overall intracellular neutrophil ROS production whereas adult athletes exhibited higher values in absolute neutrophils concentration [45]. These results suggest that children may have a different mechanism of post-exercise ROS generation by neutrophils compared to adults. However, when trained 9–11 years old boys and girls were subjected to interval type of swimming exercise protocol (12 × 50 m at an intensity of 70%–75% of their best time in 50 m with a 1-min rest period among sets), a marked decline of GSH and the ratio of reduced to oxidized glutathione (GSH/GSSG) as well as a pronounced elevation of oxidized glutathione (GSSG), TBARS and PC were observed [19]. These results suggest that an intense acute bout of a non-weight bearing exercise such as swimming increases oxidative stress in pre-adolescents and upregulates the antioxidant system in both males and females. These studies were among the first to show that exercise induced oxidative stress in the pediatric population that responds by activating antioxidant reserves and the immune system. However, a major limitation of these studies was that they did not examine the prolonged effect of exercise on oxidative stress markers (i.e., for several days after exercise). A question arises in relation to whether the development of exercise induced oxidative is transient or not.

Kabasakalis et al. [46] utilized a high intensity steady-state cardiovascular type exercise protocol of 2000 m swimming performed by adolescent male and female swimmers (15 years) either in an intermittent or a continuous manner (6 × 50 m with a 5-min rest period among sets). This work provided evidence that both modes of swimming exercise were able to elicit a transient rise of DNA damage and lipid peroxidation for several h after exercise. All oxidative stress markers returned to baseline values within 24 h of recovery whereas protein oxidation markers remained unaffected throughout the experimental period [46]. These findings were also corroborated by the more recent study of Zalavras et al. who measured oxidative stress response at three successive time points during the post-exercise period [22]. This study supports previously reported findings supporting a transient upregulation of exercise-induced oxidative stress and antioxidant activity, independent of mode and/or form.

It is uncertain if training status affects acute oxidative stress responses of children and adolescents. Tong et al. [47] showed that despite the fact that untrained adolescents (14–17 years, Tanner stages 2–4) did not demonstrate any changes in xanthine oxidase (XO), catalase and superoxidase (SOD), TAC and GSH concentrations in response to a 21-km run, they notably increased when this running trial was repeated at subsequent time points during the yearly training season. This study suggested that training may trigger an upregulation of oxidative stress responses in response to acute exercise. In agreement with these results, Zalavras et al. [22] recently, using a similar experimental design, revealed that endurance training improved not only aerobic capacity, but also increased resting TAC, and GSH reserves as well as magnified the rise of TBARS, PC and TAC and the decline of GSH in response to acute exercise (running for 45 min at 75% of VO_2max_ and at 90% thereafter until volitional exhaustion). If we consider that exercise-induced oxidative stress responses are a part of a physiological mechanism aiming to clear cellular debris in skeletal muscle after an intense exercise insult [20], training induced adaptations of the oxidative mechanism may be beneficial. These studies also demonstrate that children and adolescents may have the potential to improve the post-exercise immune response as it happens with several aspects of physical performance [29]. This is in line with the hormesis theory that states that low doses of an agent that is detrimental at high doses (e.g., acute exercise which transiently increases oxidative stress and depletes antioxidant reserves), induces an adaptive beneficial effect on the cells or organism when applied on a long-term basis (exercise training) [48].

Team sports such as football, basketball, team handball, volleyball etc., represent a sport category with unique kinesiological characteristics. Movement patterns such as acceleration, deceleration, changes of direction, jumping etc., belong to muscle explosive actions that are inherent in this type of sports. It is well documented that participation in team sports training and/or match activity induced an inflammatory response associated with muscle damage induced by the strong eccentric component of actions used in these sports [9,14,21,49,50]. This inflammatory response is always associated with a transient elevation of oxidative stress responses [6,14,21,49]. Although team sports are very popular among children and adolescents of both genders, very little is known about their effect in oxidative stress manifestations in the pediatric population. To our knowledge, the only study [51] that examined the acute effect of soccer and basketball on oxidative stress response in the youth revealed that adolescent players (16–17 years) exposed to a basketball and a soccer match demonstrated elevations in total serum peroxides, polymorphonuclear elastase and myeloperoxidase activity suggesting that team sports exert a similar effect on oxidative stress responses on adolescents as other exercise modalities.

It appears that exercise provides a powerful stimulus for the elevation of TAC (represents serum antioxidant reserves) in most studies in both trained and untrained adolescents. However, there is evidence that this response is more pronounced in the more trained individuals probably because they reach higher levels of absolute intensity [22]. However, tissue antioxidants such as GSH may be less responsive in youth compared to adults and in untrained compared to trained individuals [22]. Based on the rise of catalase and SOD activities seen in previous studies [22,47], it seems that antioxidant enzymes are responsive to exercise in the pediatric population as well. It must be noted though that there is a large variation in the exercise testing modes used in the literature. For example, Zalavras et al. [22] used a time-trial whereas others used tests characterized by a more progressive rise in intensity. Due to the fact that redox responses are dependent upon the extent of mitochondrial utilization and that of xanthine and NADPH oxidase during and following exercise [17], the mode as well as the intensity of exercise may play an important role in these responses (cardiovascular vs. speed/power modes). Another confounding factor in studies examining exercise-induced oxidative stress responses in children and adolescents may be the time of sampling since there is data indicating a time-dependent response [17]. Moreover, it would be interesting to determine whether a threshold-intensity exists in respect to oxidative stress responses in children and adolescents. Future studies should also examine oxidative stress responses at the same absolute vs. the same relative intensity in trained and untrained children and adolescents.

A confounding factor that should be taken into account by future investigations is the body weight status of children and adolescents. Obesity has been shown to cause a reduced antioxidant ability in the adult population [52,53,54], thereby predisposing or favoring a higher systemic oxidative stress in the obese compared to individuals of normal weight [52]. It appears that this may be the case in children as well. It has been reported that resting levels of pro-oxidation biomarkers were greater and anti-oxidation biomarkers were lower in obese compared to normo-weight pre- and early-pubertal children [39,55]. Moreover, it has been shown that body mass index (BMI) is positively correlated with pro-oxidation markers and negatively with anti-oxidation markers in children [56]. These findings are in agreement with previous observation suggesting that low-grade systemic inflammation due to excessive adipose tissue stores may result in reduced antioxidant reserves and thus increased generation of RONS [57]. However, until recently, we were unaware if this obesity-related rise in baseline oxidative stress can affect exercise-induced responses in the pediatric population. Paltoglou et al. [39] showed that obese pre-pubertal boys (10–11 years, puberty was determined using measurements of testosterone concentration) demonstrate a greater exercise-related response of oxidative stress biomarkers compared to their normal-weight counterparts most probably due to a lower antioxidant capacity. In that study, waist circumference was found to be the best negative predictor of glutathione peroxidase activity (GPX) and the reduction of GSH post-exercise.

In conclusion, it appears that children and adolescents, similarly to adults, demonstrate an elevation of pro-oxidant markers which is associated with a parallel upregulation of their antioxidant activity. These responses seem to be of a lower magnitude in youth compared to adults. Pre-pubertal children may also have lower antioxidant reserves than adults. When children are contrasted to adults, it appears that children may be more susceptible to a rise in RONS in response to exercise than adults due to their faster kinetics of oxygen consumption (VO_2_) as well as the greater elevation of neutrophils in the circulation post-exercise [37]. This is surprising since children demonstrate exercise-induced muscle damage of smaller magnitude compared to adults and as such a lower inflammatory response [58,59]. However, more recent data have shown that adults demonstrate greater oxidative stress responses than adolescents [22], a finding that coincides with others suggesting important antioxidant molecules such as GSH could be less responsive in youth compared to adults. Moreover, children may also exhibit a different pattern of neutrophil mobilization in response to damaging exercise compared to adults. IL-6 response to exercise has been shown to be associated with neutrophil cell post-exercise indicating that neutrophil mobilization may be related to changes in this cytokine [45]. It appears that boys (8–10 years), compared to men, demonstrated a rise in protein carbonyl concentration in the circulation post-exercise and increased their total intracellular neutrophil ROS generation whereas adult males did not [45]. In contrast, boys’ total neutrophil counts increased in adult males post-exercise but not in boys suggesting that exercise elicits a greater recruitment of leukocytes in men compared to boys [45]. These findings coincide with previous reports of a smaller neutrophil response in children compared to adults post-exercise [60,61]. It has also been speculated that that in boys, neutrophil mobilization in response to exercise may originate from the bone marrow and not from the circulation [45]. Overall, these data suggest that children may be more prone to free radical generation and as such more sensitive to changes in their redox status in response to exercise despite a smaller skeletal muscle damage and neutrophil mobilization. This response may partly be explained by increases of growth hormone following acute exercise [62,63,64] which seems to be implicated in neutrophil mobilization since in vitro studies have identified its receptors on these immune cells [65]. One may argue that growth hormone increase following exercise is higher in adults in absolute terms, however children experience a greater relative growth hormone rise from its resting values [45], probably as a part of the overall anabolic mechanism supporting their growth and development [61]. It is possible that differences observed in the overall inflammatory response as well as in redox status perturbations with exercise may be related to adaptations associated with growth observed during pre- and early-puberty. If so, studying of redox adaptations of children and early adolescents to exercise compared to adults may promote our knowledge of growth and development in the pediatric population. Table 1 lists all studies that examined oxidative stress responses of children and adolescents to various types of acute exercise.

## 4. Responses to Chronic Exercise

Children and adolescents demonstrate an extensive participation in sport activities mostly characterized by a short duration and intermitted pattern [66,67]. It is most likely to participate in repetitive training sessions and competition events on a daily basis and for prolonged periods of time. Exercise training has been shown to promote a number of positive health- and performance-related adaptations in both adults and children [68,69,70]. It would be very interesting to know whether the pro-oxidant and, more importantly, the anti-oxidant mechanism respond to a training stimulus in the pediatric population. An improved antioxidant capacity would lead to reduced oxidative stress at rest and in response to exercise, an adaptation which would enhance children’s ability to boost their immune system, improve their performance and upregulate their recovery potential.

Only a few studies have examined adaptations of the pro- and anti-oxidant mechanisms in children and adolescents in response to systematic exercise training. Swimming was employed by two of these studies. In the study of Kabasakalis et al. [71] the effect of 23 weeks of swimming training on oxidative stress in 10–11 years old boys and girls (Tanner stages 1 and 2) was investigated. They reported that, although systemic levels of TAC and TBARS remained unaltered, training increased the GSH/GSSG ratio due to an elevation of GSH stores suggesting an enhancement of antioxidant system. This upregulation of the antioxidant capacity coincided with an improvement of swimming performance. On the other hand, controversial results found in another study with children (~10 years) of similar characteristics who were also exposed to swimming training [33]. In that study the authors concluded that young swimmers demonstrated higher values on oxidative markers, such as TBARS, and lower values on antioxidant markers, such as catalase activity and GSH/GSSG in comparison to control subjects [33]. However, that study was actually a cross-sectional study with no training intervention applied and athletes had a slightly higher maximal oxygen consumption than age-matched controls. The different results from these two studies indicate that other factors which were not investigated may have a significant role on oxidative stress responses in response to training. Perhaps the total training load in a given training cycle, participants’ initial fitness level (in the study by Gougoura et al. control children had a maximal oxygen consumption that exceeded 48 mL/kg/min) and even the social life as well as other daily activities could influence children’s response to training.

An attempt to investigate the relation between fitness level and oxidative stress responses after a 20-m multistage incremental test (20-mSRT) was made by another cross-sectional study [72]. A total of 132 Spanish prepubertal (pre-puberty was determined using measurements of sex hormone concentrations in blood) boys and girls (7–12 years) volunteered to participate in this study. Blood samples were taken at rest and then children participated in 20-mSRT. Authors assigned children to either a higher or lower fitness level subgroups. It was reported that well-conditioned children demonstrated favorable adaptations in antioxidant system in comparison to those with lower physical fitness or those who were sedentary [72]. In fact, a positive association between cardiorespiratory fitness and the ratio of reduced to oxidized glutathione was observed suggesting that an enhancement of cardiovascular is related to a reduction of oxidative stress.

When an attempt was made to examine the effects of total training load, results were highly inconclusive. The main objective of a cross-sectional study performed by Santos-Silva et al. [34] was to investigate the effect of large training load on oxidative stress responses using swimming as well. In this study, 40 high competition swimmers aged 12–16 years participated and trained for more than 40 h/week. Control age-matched adolescents participated only in physical education classes in school for 2–4 h/week. Swimmers exhibited a higher TBARS and TBARS/TAC ratio than controls. The authors concluded that participation in demanding training of high volume may lead to high levels of ROS. However, time of sampling may have influenced these results since it has been shown that oxidative stress biomarkers remain elevated for as long as 5–7 days following intense exercise, at least in adults [2,14,16]. Performance was not measured in that study. In contrast, Tong et al. [73] showed that chronic endurance exercise training of adolescent athletes (12–16 years, sexual maturation was not assessed) with cycling and running resulted in higher values of XO, GSH and catalase activity compared to controls suggesting a training-induced upregulation of the antioxidant system (although TAC and SOD remained unaltered). This upregulation of the antioxidant system in that study coincided with an increase of endurance performance. In line with these results, a recent study demonstrated that a year-long training program of running not only improved all measures of endurance performance but also increased anti-oxidation and lowered pro-oxidation in both adult and adolescent (14–15 years, Tanner stages 2–3) participants involved in training compared to age-matched controls [22]. Trained adolescents had lower values of PC and TBARS and an attenuated decline of TAC and GSH in response to acute exercise compared to controls. Authors concluded that untrained adolescents may have a lower antioxidant reserve [22]. In line with these findings, another study reported that a 3-year long training period with gymnastics increased performance and the activity of glutathione peroxidase but decreased the activity of superoxide dismutase when compared to control [74].

There is very limited information on the effects of team sports on oxidative stress response in the pediatric population. Team handball training has been shown to enhance SOD activity and cause a positive correlation between aerobic capacity and catalase activity as well as a negative one between aerobic capacity and hydrogen hyperoxide in ~17-year-old subjects [75]. Similarly, according to a cross-sectional study, basketball players demonstrated higher values of TAC compared to controls [76]. However, in that study, a higher antioxidant status of basketball players did not translate into lower values of oxidative stress markers. A comparison between adolescent football players and control participants (12–13 years old) before and after a period of six months of training revealed that the former had a higher antioxidant capacity than the later [77]. These results suggest that team sports are likely to cause favorable adaptations in respect to pro- and anti-oxidation in the pediatric population as well.

Collectively, these results suggest that there is evidence that exercise training induces a positive adaptation in oxidative stress responses in the pediatric population by elevating antioxidant capacity and as such reducing systemic levels of oxidative stress. However, more studies using training interventions are needed. Future research should explore the optimal training characteristics (intensity, duration, frequency) for such positive responses for various exercise modalities. Table 2 lists all studies that examined oxidative stress responses of children and adolescents to various types of chronic exercise.

## 5. Physiological Significance

Adolescence is a crucial period during maturation in humans because it is associated with significant hormonal perturbations that affect not only the individual’s sexual and reproductive characteristics but also affect musculoskeletal characteristics [78]. This period is characterized by dramatic alterations in the regulation of hypothalamic-growth hormone (GH)–insulin-like growth factor (IGF)-1 and hypothalamic–pituitary–gonadal axes [79]. It has also been reported that GH deficiency in children is associated with reduced antioxidant capacity whereas GH replacement therapy reverses this phenomenon [80]. Similarly, GH deficiency in adults is accompanied by endothelial dysfunction and elevated oxidative stress which is attenuated by GH replacement therapy [81]. Moreover, reduced physiological levels of testosterone coincide with an altered capacity of immune cells to produce ROS [82]. In support of this observation, elevated circulating testosterone in adolescents demonstrates a negative association with oxidative stress markers [83]. These findings suggest that antioxidant capacity seems to mature with age, especially during the transition from pre-adolescence to early puberty and this may also be true for exercise-induced responses of oxidative stress biomarkers [39,44]. Exercise does not only represent a potent stimulus for mitochondrial substrate metabolism for energy release that causes increased ROS generation but also activates the GH axis during puberty [56,84,85]. Therefore, basal and exercise-induced oxidative stress adaptations in the pediatric population are seen during periods of physiological maturation, probably during the transition from pre-puberty to early puberty, a process that may be disrupted in cases of hormonal deregulation. As such, exercise training may aid this transition by causing favorable adaptation to the anti- and pro-oxidant state of children. Furthermore, exercise’s favorable effects on obesity status may be also be related to an attenuation of pro-oxidation and enhancement of anti-oxidation. Evidence of an inhibition of GH secretion at pituitary and hypothalamus in response to augmented levels of insulin, free IGF1 and free fatty acid concentrations further supports this notion [86]. Exercise stimulates GH release probably via the JAK2-STAT5 phosphorylation pathway [87,88] and it has been shown to correlate positively with antioxidant status biomarkers in pre-adolescents with normal weight but not with the obese ones [89]. In fact, it was reported that resting GH concentration was the best negative predictor for post-exercise PC response and the exercise-induced elevation of GH was positively correlated with GSSG rise post-exercise and negatively with PC [39] suggesting a close association between redox status and maturation.

It is characteristic that a 3-fold increase in RONS has been seen in STAT5A knock-out cells [90]. Moreover, GSH synthesis and/or the activities of various antioxidant enzymes (e.g., GSH reductase and GPX) are mediated via intracellular pathways related to STAT5 expression [91]. It is characteristic that prolonged GH administration to obese animals inhibited lipid peroxidation markers in fat cells and upregulated the expression of antioxidant enzymes [92]. On the other hand, GH deficiency in children and adults has been associated with elevated oxidative stress markers, a response that is reversed following with GH replacement treatment [80,81].

These findings suggest that exercise is capable of stimulating pro- and anti-oxidant responses during pre- and post-puberty independent of body weight status. However, it appears that entry into puberty coincides with an improvement of antioxidant activity probably due to the augmented production and release of GH and androgens. Obesity appears to decrease antioxidant capacity and thus increase exercise-induced oxidative stress in children and adolescents probably due to a GH-dependent mechanism, further highlighting the negative implications of obesity. Whether the transition from pre-adolescence to early adolescence contributes to the maturation of antioxidant mechanisms warrants further investigation by future studies. This possibility should be explored in relation to fatness level.

## 6. Conclusions

The present review attempted to present all available scientific evidence regarding the acute and chronic effects of exercise on oxidative stress responses in the pediatric population. Most studies support the idea that acute exercise may induce a relevant, but transient increase in oxidative markers. It appears that these exercise-induced responses may be dependent on children’s maturity stage and body weight status. Other factors that may affect the magnitude of these responses are fitness status, the total training load and the type of activity. Duration, intensity and frequency of exercise may also play a significant role.

## Figures and Tables

**Table 1:** Acute effects of exercise on oxidant and antioxidant markers.

Study	Exercise	Subjects	Tissue	Oxidant and Antioxidant Markers
Nikolaidis et al. (2007) [19]	Swimming Volume: 50 m × 12 reps. Intensity: 70%–75% of best time Rest periods: 1 min	11 boys and 11 girls, 9–11 years old Training age >1 year Training frequency >3 times/week	Serum	CAT↑, GSH↓, TBARS↑, PC↑, TAC↑
Zalavras et al. (2015) [22]	Time trial, 45 min in ~75% of VO_2max_ and until exhaustion in 90% of VO_2max_	13 trained adolescents, 11 untrained adolescents, 12 trained adults, 10 untrained adults. Pre-intervention, at mid of macrocycle and at the end of macrocycle	Serum, erythrocytes	TAC ↑ (excepted the trained adolescents in first trial), GSH ~ (athletes) and ↓ in non-athletes, CAT↑, TBARS ↑, PC↑, UA↑ Bilirubin ↑
Paltoglou et al. (2015) [39]	Time trial in 70% of VO_2max_ until exhaustion	76 healthy normal weight and obese children (pre-pubertal and pubertal)	Serum, erythrocytes	TBARS↑, PC↑, GSH↓, GSSG↑, GSH/GSSG↑, GPx↑, TAC↑, CAT↑ (excepted the normal weight in early pubertal children)
Benites-Sillero et al. (2009) [44]	20 m-Shuttle run test incremental exercise test	38 prepubescent and 32 pubescent non-athlete boys	Saliva	GSH↑
Liu and Timmons (2015) [45]	Volume: 2 × 30 min, Rest period: 6 min rest Intensity: 60% VO_2max_	10 children 8–10 years old 12 adults 19–21 years old	Blood	Children Absolute Neutrophils ~, Intracellular Neutrophils↑, PC ~, MDA ~
Kabasakalis et al. (2014) [46]	Swimming 2000 m 50 m × 6 reps, Intensity: maximal, rest periods: 5 min	15 boys and 15 girls, 14–18 years old Training age >1 year Training frequency >3 times/week	Plasma CSH in Erythrocytes	2000 m 8-OHdG ↑PC~, MDA↑, GSH↓, UA ~, Bilirubin↓ (post), Bilirubin ↑ (24 h post) 6 × 50 m 8-OHdG ↑PC~, MDA↑, GSH↓ (24 h), UA↑, Bilirubin↓ (1 h post), Bilirubin↑ (24 h post)
Tong et al. (2013) [47]	Endurance 21 km	10 runners 14–17 years old, one year study. Acute effects were examined two times pre- and post-one year training	Serum	Pre-intervention TBARS↓, XO ~, CAT ~, GSH ~, SOD↓, TAC ~ Post intervention TBARS↓, XO↑, CAT↑, GSH~, SOD ↓, TAC ~
Pereea et al. (2015) [51]	Basketball and soccer game	35 football players and 13 basketball players 16–17 years old	Serum,	Football TSPs↓, PMN elastase↑, MPO~, Fibrinogen ~ Basketball TSPs↑, PMN elastase↓, MPO~, Fibrinogen ~

CAT: catalase activity, TAC: total antioxidant capacity, TBARS: thiobarbituric acid-reactive substances, MDA: Malondialdehyde, 8-OHdG: DNA damage marker, PC: protein carbonyls, TG: total glutathione, GSH: reduced glutathione, GSSG: oxidized glutathione, GSH/GSSG: reduced to oxidized glutathione form ratio, GPx: glutathione peroxidase, CAT: catalase, SOD: superoxide oxidase, XO: xanthine oxidase, UA: uric acid, TSPs: serum total peroxide, PMN elastase: polymorphonuclear elastase, MPO: myeloperoxidase; ~ denotes approximately; ↓: denotes a decline of a marker; ↑: denotes an increase of a marker.

**Table 2:** Chronic adaptations of exercise on oxidant and antioxidant markers.

Study	Exercise	Subjects	Tissue	Oxidant and Antioxidant Markers
Zalavras et al. (2015) [22]	Endurance training measures during the course of a training cycle	13 trained adolescents, 11 untrained adolescents, 12 trained adults, 10 untrained adults. Pre-intervention, at mid of macrocycle and at the end of macrocycle	Serum, erythrocytes	Trained adolescents demonstrated PC↓, TBARS↓ and an attenuated decline of TAC and GSH in response to acute exercise compared to controls
Gougoura et al. (2007) [33]	Swimming comparison between athletes and controls	17 athletes 10–12 years old, training age >1 year, training frequency 3 times/week, duration 1 h	Serum Blood	GSH↑, GSSG ~, GSH/GSSG↓, TBARS↑, TAC↓, CAT↓, UA~
Santos-Silva et al. (2001) [34]	Swimming	40 high level athletes 12–16 years old, training duration 20 h/week	Plasma	TAS ~, TBARS ↑
Kabasakalis et al. (2007) [71]	13–23 weeks of swimming training	24 boys and girls 10–11 years old, training age >1 year, training frequency >3 times/week, duration 75–90 min	Plasma Erythrocyte	TBARS ~, PC↑, GSH↑, GSSG↓, GSH/GSSG↑, TAC~, CAT~
Llorente-Cantarero FJ et al. (2012) [72]	Status, rest values among children with different training status	132 boys and girls 7–12 years old	Plasma Erythrocyte	Children with pure training status demonstrated TG↑, GSSG↑, GSH/GSSG↓
Tong et al. (2012) [73]	Endurance athletes	67 male runners, cyclists and untrained adolescents	Serum	Cyclists demonstrated higher values in XO, GSH AND CAT in comparison to runners and controls Runners demonstrated higher values in CAT in comparison to controls
Alshammari et al. (2010) [74]	Comparison between trained and untrained children, 3 years training period	38 girls, 11 years old training duration >10 h per week,	Serum	GPx was higher and SOD was lower in trained children
Djordgevic et al. (2011) [75]	Handball training	33 handball players and untrained individual, 16–19 years old and training age 7–10 years	Plasma Erythrocyte	Athletes demonstrated higher activity in SOD, lower activity in CAT and higher level in TBARS concentration
Yilmaz et al. (2007) [76]	Basketball training	Adolescent basketball players	Serum, plasma	TAC was higher in basketball players Oxidative stress index and total peroxide level did not differ between group
Zivkoviz et al. (2013) [77]	Soccer training 6 months intervention	26 male soccer players and 26 non-athletes, 12–13 years old	Blood samples	After 6 months training the following alterations were occurred TBARS↑, SOD↑, CAT↑, GSH↓, H_2_O_2_~, O_2_−~

TAC: total antioxidant capacity, TAS: total antioxidant status, TBARS: thiobarbituric acid-reactive substances, PC: protein carbonyls, TG: total glutathione, GSH: reduced glutathione, GSSG: oxidized glutathione, GSH/GSSG: reduced to oxidized glutathione form ratio, GPx: glutathione peroxidase, CAT: catalase, SOD: superoxide oxidase, XO: xanthine oxidase, UA: uric acid, H_2_O_2_: hydrogen peroxide, O_2_−: superoxide anion radical; ~ denotes approximately; ↓ denotes a decline of a marker: ↑: denotes an increase of a marker.
